# Unsupervised primaquine for the treatment of *Plasmodium vivax* malaria relapses in southern Papua: A hospital-based cohort study

**DOI:** 10.1371/journal.pmed.1002379

**Published:** 2017-08-29

**Authors:** Nicholas M. Douglas, Jeanne Rini Poespoprodjo, Dewi Patriani, Michael J. Malloy, Enny Kenangalem, Paulus Sugiarto, Julie A. Simpson, Yati Soenarto, Nicholas M. Anstey, Ric N. Price

**Affiliations:** 1 Global and Tropical Health Division, Menzies School of Health Research, Charles Darwin University, Darwin, Northern Territory, Australia; 2 Division of Infectious Diseases, Christchurch Hospital, Christchurch, New Zealand; 3 Timika Malaria Research Program, Papuan Health and Community Development Foundation, Timika, Papua, Indonesia; 4 Department of Child Health, Faculty of Medicine, University Gadjah Mada, Yogyakarta, Indonesia; 5 Centre for Epidemiology and Biostatistics, Melbourne School of Population and Global Health, University of Melbourne, Melbourne, Victoria, Australia; 6 Victorian Cytology Service Ltd., Melbourne, Victoria, Australia; 7 Mimika District Hospital, Timika, Papua, Indonesia; 8 Rumah Sakit Mitra Masyarakat, Timika, Papua, Indonesia; 9 Centre for Tropical Medicine and Global Health, Nuffield Department of Clinical Medicine, University of Oxford, Oxford, United Kingdom; Liverpool School of Tropical Medicine, UNITED KINGDOM

## Abstract

**Background:**

Primaquine is the only licensed drug for eradicating *Plasmodium vivax* hypnozoites and, therefore, preventing relapses of vivax malaria. It is a vital component of global malaria elimination efforts. Primaquine is efficacious when supervised in clinical trials, but its effectiveness in real-world settings is unknown. We aimed to determine whether unsupervised primaquine was effective for preventing re-presentation to hospital with vivax malaria in southern Papua, Indonesia.

**Methods and findings:**

Routinely-collected hospital surveillance data were used to undertake a pragmatic comparison of the risk of re-presentation to hospital with vivax malaria in patients prescribed dihydroartemisinin-piperaquine (DHP) combined with primaquine versus those patients prescribed DHP alone. The omission of primaquine was predominantly due to 3 stock outages. Individual clinical, pharmacy, and laboratory data were merged using individual hospital identification numbers and the date of presentation to hospital. Between April 2004 and December 2013, there were 86,797 documented episodes of vivax malaria, of which 62,492 (72.0%) were included in the analysis. The risk of re-presentation with vivax malaria within 1 year was 33.8% (95% confidence Interval [CI] 33.1%–34.5%) after initial monoinfection with *P*. *vivax* and 29.2% (95% CI 28.1%–30.4%) after mixed-species infection. The risk of re-presentation with *P*. *vivax* malaria was higher in children 1 to <5 years of age (49.6% [95% CI 48.4%–50.9%]) compared to patients 15 years of age or older (24.2% [95% CI 23.4–24.9%]); Adjusted Hazard Ratio (AHR) = 2.23 (95% CI 2.15–2.31), *p* < 0.001. Overall, the risk of re-presentation was 37.2% (95% CI 35.6%–38.8%) in patients who were prescribed no primaquine compared to 31.6% (95% CI 30.9%–32.3%) in those prescribed either a low (≥1.5 mg/kg and <5 mg/kg) or high (≥5 mg/kg) dose of primaquine (AHR = 0.90 [95% CI 0.86–0.95, *p* < 0.001]). Limiting the comparison to high dose versus no primaquine in the period during and 12 months before and after a large stock outage resulted in minimal change in the estimated clinical effectiveness of primaquine (AHR 0.91, 95% CI 0.85–0.97, *p* = 0.003). Our pragmatic study avoided the clinical influences associated with prospective study involvement but was subject to attrition bias caused by passive follow-up.

**Conclusions:**

Unsupervised primaquine for vivax malaria, prescribed according to the current World Health Organization guidelines, was associated with a minimal reduction in the risk of clinical recurrence within 1 year in Papua, Indonesia. New strategies for the effective radical cure of vivax malaria are needed in resource-poor settings.

## Abstract 2

In a pragmatic hospital-based cohort study, Ric Price and colleagues compared risk of re-presentation with vivax malaria in patients prescribed dihydroartemisinin-piperaquine combined with primaquine versus patients who could not be given primaquine due to stock outages.

## Author summary

**Why was this study done?:**

- *Plasmodium vivax* forms dormant liver stages (hypnozoites) that can cause relapsing illness weeks to months after the initial infection.
- Preventing these relapses has major public health benefits by reducing clinical morbidity and lowering the risk of parasite transmission.
- A 14-day course of primaquine (the only licensed drug that is active against hypnozoites) has been previously shown to have greater than 90% efficacy for preventing relapses, but its effectiveness in nonstudy settings has never been tested and may be substantially lower due to nonadherence to necessarily long treatment regimens.

**What did the researchers find?:**

- Three stock outages of primaquine at a hospital in Papua, Indonesia enabled us to assess whether prescription of primaquine in combination with dihydroartemisinin-piperaquine (DHP) for vivax malaria was associated with a lower rate of subsequent re-presentation to hospital with vivax malaria compared to those patients prescribed DHP alone.
- Between 2006 and 2013, there were 62,492 patient presentations with clinical vivax malaria that were included in analyses, 87% of which were treated with primaquine.
- Overall, one third of patients re-presented to hospital with vivax malaria within 1 year; those prescribed primaquine being only 10% less likely to re-present than those who did not receive primaquine.

**What do the findings mean?:**

- The results of this study suggest that primaquine is substantially less effective for preventing relapses of vivax malaria in real-world practice than is predicted by clinical efficacy trials and that this is a likely consequence of incomplete adherence to treatment.
- Efforts to improve adherence to primaquine and to develop alternative drugs with shorter dosing regimens and greater patient tolerability are needed to achieve the significant public health benefits of the radical cure of *P*. *vivax*.

## Introduction

Primaquine is the only licensed drug known to be active against *Plasmodium vivax* hypnozoites. It is critically important for preventing relapses and thus reducing the global burden of vivax malaria [1]. The World Health Organization (WHO) recommends a total dose of 3.5–7 mg/kg primaquine divided over 14 days for the treatment of vivax malaria in those who are not deficient in the glucose-6-phosphate dehydrogenase (G6PD) enzyme [2]. Outside of clinical trials, adherence to this long regimen is commonly believed to be poor [3, 4]. Prospective studies of treatment supervision have yielded inconsistent results [5–8] and are challenging to interpret since study involvement itself tends to have an effect on treatment compliance [9]. Despite widespread use since the 1960s, there have not been any rigorous studies of the effectiveness of prescribing primaquine in routine practice in vivax-endemic regions.

Many National Malaria Control Programs have been reluctant to advocate primaquine for the radical cure of *P*. *vivax* infections because of the risk of inducing severe hemolysis in G6PD-deficient patients and the difficulty of promoting practical pretreatment testing to reduce exposure to those individuals at greatest risk [10]. However, the risk of drug toxicity needs to be balanced against the cumulative morbidity caused by recurrent *P*. *vivax* relapses arising from the dormant liver stages of the parasite [11–13]. Recent guidelines from WHO advocate that, where possible, all patients should be tested for G6PD deficiency prior to the administration of primaquine; however, few malaria-endemic countries have incorporated this into clinical practice.

We aimed to determine the effectiveness of primaquine in combination with dihydroartemisinin-piperaquine (DHP), prescribed according to national policy without prior testing for G6PD deficiency, for preventing recurrent *P*. *vivax* malaria. Three stock outages of primaquine at a hospital in Papua, Indonesia provided an opportunity for a pragmatic comparison of the risk of re-presentation to hospital with vivax malaria in patients treated with DHP plus primaquine compared to those treated with DHP alone.

## Methods

### Ethical approval

Ethical approval for this study was obtained from the Health Research Ethics Committees of the University of Gadjah Mada (KE/FK/544/EC), Indonesia and Menzies School of Health Research, Darwin, Australia (HREC 10.1397).

### Study design

This was a retrospective cohort study which used routinely-collected hospital surveillance data to determine the comparative rate of re-presentation to hospital with vivax malaria in patients treated with DHP with or without primaquine. The analysis followed an a priori statistical plan that is available in S1 Text. The RECORD checklist is provided in S2 Text.

### Study site, hospital characteristics, and treatment protocols

The geography, climate, and demographics of Mimika District and its capital, Timika, have been described previously [14, 15]. Briefly, this region lies in south-central Papua, Indonesia. Timika has an expanding population—currently approximately 200,000 people—of whom half are native highland or lowland Papuans, and half are migrants from elsewhere in Indonesia [15].

Malaria transmission is perennial with minimal seasonal variation. In 2013, the point prevalence of parasitemia was estimated to be 13.9%, 46% due to *P*. *vivax* and 2% due to mixed *P*. *falciparum/P*. *vivax* infections [16]. Local *P*. *vivax* strains have a typical equatorial relapse interval of 3–4 weeks [17, 18] and are highly resistant to chloroquine [19–21].

Rumah Sakit Mitra Masyarakat (RSMM) is the largest health care facility in Timika and one of two public hospitals in the district. The other hospital opened in 2008, and since then, RSMM has received an estimated 80% of malaria patients attending either of the two hospitals. RSMM has an active outpatient department, a 24-hour emergency department, four wards with 110 beds for inpatient care, and a “high care” facility.

DHP replaced quinine and chloroquine as the first-line treatment of uncomplicated malaria due to any *Plasmodium* species at RSMM in March 2006. An unsupervised, 14-day course of primaquine was also recommended for the treatment of patients with *P*. *vivax* mono- or mixed infections with a change in policy from low-dose (3.5 mg/kg total dose) to high-dose (7 mg/kg) primaquine in March 2006, coinciding with the policy change for blood schizontocidal treatment. In the outpatient clinic, primaquine is commenced at the same time as schizontocidal treatment. For inpatients, primaquine is administered once clinical recovery has begun and patients are able to take oral medication.

Based on a local prevalence survey, 2.6% of the population have at least intermediate G6PD deficiency (16). Testing for G6PD deficiency prior to prescribing primaquine is not done routinely. Patients with very dark urine, potentially indicative of severe hemolysis, are advised to stop taking primaquine and return to the hospital for clinical review.

### Laboratory and data collection procedures

All patient presentations to RSMM between 2004 and 2013 were recorded in a Q-Pro database by hospital administrators. Details entered for each presentation included individual hospital identification number, demographic details, dates of presentation and discharge, and clinical diagnoses assigned by the attending physician. Details of all prescriptions at the hospital pharmacy were also recorded in a separate database.

Strict hospital protocols required that all patients presenting to the outpatient department with fever or other symptoms potentially compatible with malaria and all inpatients, regardless of clinical picture, had a blood smear taken for Giemsa staining and examination under a light microscope. In most cases, parasitological diagnosis was made from a thick film. In a minority of cases, thin films were also read. Rapid diagnostic tests based on the Histidine Rich Protein-2 (HRP2) antigen were used occasionally to confirm *P*. *falciparum* infection.

### Data preparation

Clinical data for each patient presentation were merged with antimalarial prescription data using individual hospital identification numbers and dates. Data were concatenated such that multiple presentations with malaria within 14 days of the initial presentation were grouped as a single “episode,” the rationale being that recurrent parasitemia due to reinfection or relapse is highly unlikely within this period [18].

Total primaquine doses were estimated in milligrams per kilogram from the numbers of tablets provided by the pharmacy and the predicted weight of each patient, which was derived from the mean weight of age-, sex-, and ethnicity-matched patients in a local cross-sectional survey [15]. Prescribed doses were then categorized as: (a) no primaquine (matching antimalarial prescription data but no primaquine prescribed), (b) single-dose primaquine (<1.5 mg/kg), (c) low-dose primaquine (≥1.5 mg/kg and <5 mg/kg), (d) high-dose primaquine (≥5 mg/kg), or (e) unknown dose (matched primaquine prescription record but unable to determine dose in mg/kg—mostly due to nonsensical dose fractions).

### Statistical analysis

Recurrent malaria following *P*. *vivax* infection can be due to: (i) recrudescence of a partially treated infection (which usually occurs early and has been accounted for in this analysis by concatenating clinical episodes into 14-day events), (ii) reinfection in patients continuing to reside in a malaria-endemic area, or (iii) relapse from reactivation of the dormant hypnozoite stage. In Papua and other equatorial regions, relapses start to occur 21 days after the initial infection and can potentially continue for over a year. An individual will therefore be at excess risk of vivax malaria over and above the baseline risk of reinfection whilst the relapses continue to occur.

**Fig 1:**
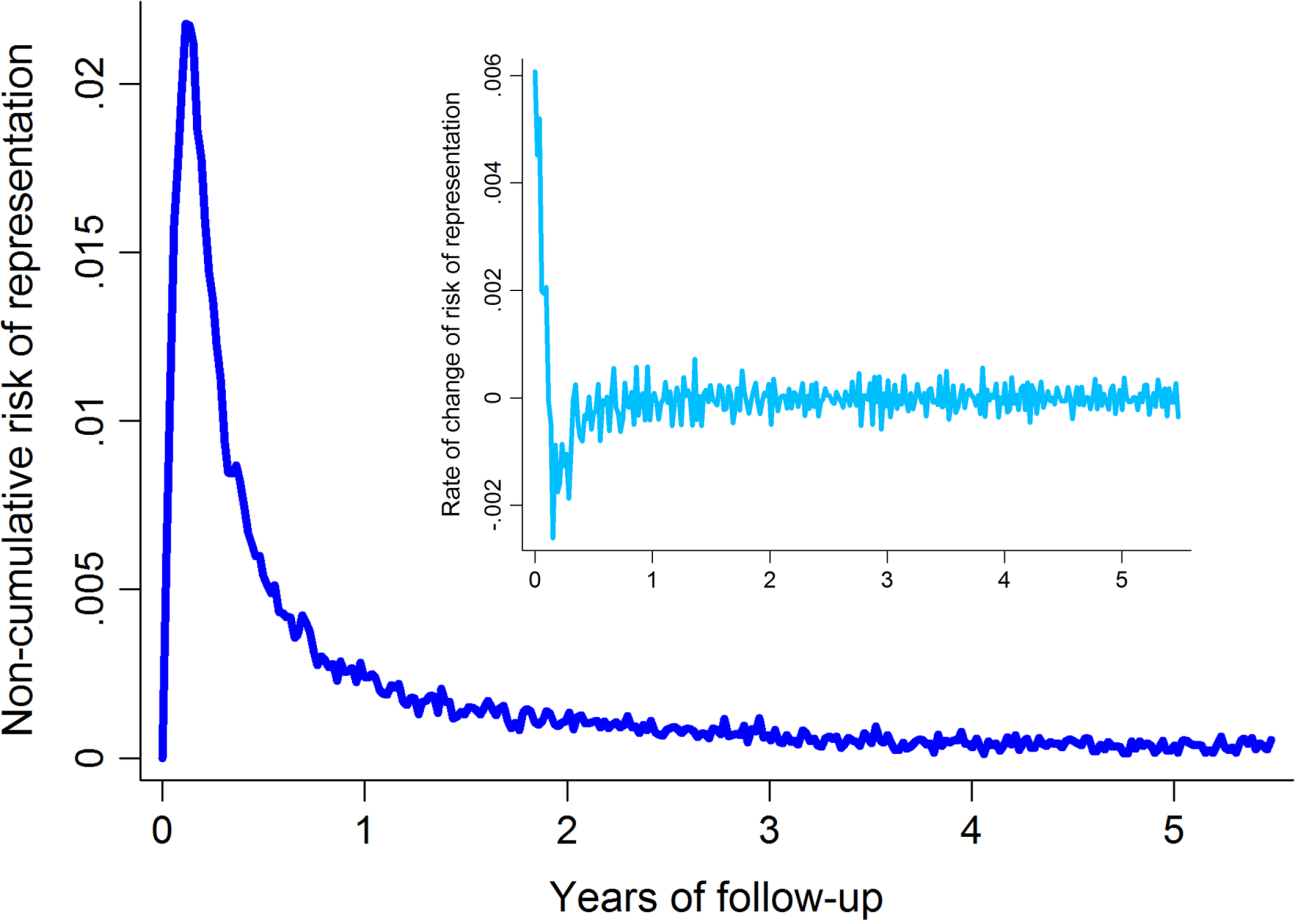
Noncumulative risk of re-presentation to hospital with vivax malaria following initial *P*. *vivax* infection (main figure) and the rate of change of the risk of re-presentation (inset).

In order to determine the optimal duration of patient follow-up that would capture the excess rate of re-presentation due to relapse whilst minimizing the influence of background reinfection rates, graphs of the first and second derivatives of the overall failure curve were generated (Fig 1). These provided graphical representation of the noncumulative risk of re-presentation by time and the rate of change in risk of re-presentation by time, respectively. The point of inflexion of the first derivative and the point at which the second derivative settled at 0 represent the time at which the baseline risk of presentation with vivax malaria is reached; this occurred at about 1 year after the initial episode of *P*. *vivax* malaria (Fig 1). Follow-up data were therefore censored at 365 days.

To ensure comparability of patients in this study, only episodes of *P*. *vivax* malaria that were treated with DHP as the blood schizontocidal agent were included. As hospital treatment policy precluded treatment of pregnant women and infants aged less than 1 year with primaquine, these individuals were excluded from analyses. The primary comparison was between patients treated without primaquine and those treated with either a low or high total dose of primaquine—in other words, with a potentially curative regimen. All prespecified subgroup analyses were limited to patients receiving either no primaquine or high-dose primaquine (hospital protocol) and included: [1] individual age categories (1 to <5 years, 5 to <15 years, and ≥15 years), [2] patients initially treated as outpatients, [3] patients treated during, or 12 months before and after, the largest primaquine stock outage from July 2007 to February 2008, and [4] patients prescribed either no primaquine or a total primaquine dose greater than 7 mg/kg. Since a longer period of follow-up will result in a greater proportion of recurrent episodes of vivax malaria attributable to reinfection, subanalyses were also conducted in which follow-up was restricted to 3 months; during this period, relapsing infections will have constituted the majority of recurrences. All analyses were performed in STATA version 12.1 (College Station, Texas). Kaplan-Meier failure curves for the risk of re-presentation to hospital with *P*. *vivax* infection were plotted, stratified by the baseline characteristics sex, pregnancy status, ethnicity (non-Papuan, Highland Papuan, Lowland Papuan), age group (as above), year of initial presentation, presence or absence of *Plasmodium* coinfection, episode number (first through fifth), initial admission status (outpatient or inpatient), and primaquine dose category.

Cox proportional hazards regression was used for analyses of primaquine effectiveness. The proportional hazards assumption was assessed by visually comparing the log(cumulative hazard) by time of follow-up curves for each covariable category and subsequently by fitting and comparing models with and without time of follow-up interaction terms. All multivariable models were stratified by year to control for changes in the background malaria endemicity over the 8-year study period.

Approximately 40% of patients in the database had multiple episodes of vivax malaria. Up to 5 episodes per individual were included in the Cox models, and the variance–covariance matrices were corrected for intraindividual correlation using robust standard errors. Since number of episodes was predictive of a further presentation, this was also included as a covariable in the final models. Kaplan-Meier estimates were based on the risk of recurrence after the first episode only.

## Results

**Fig 2:**
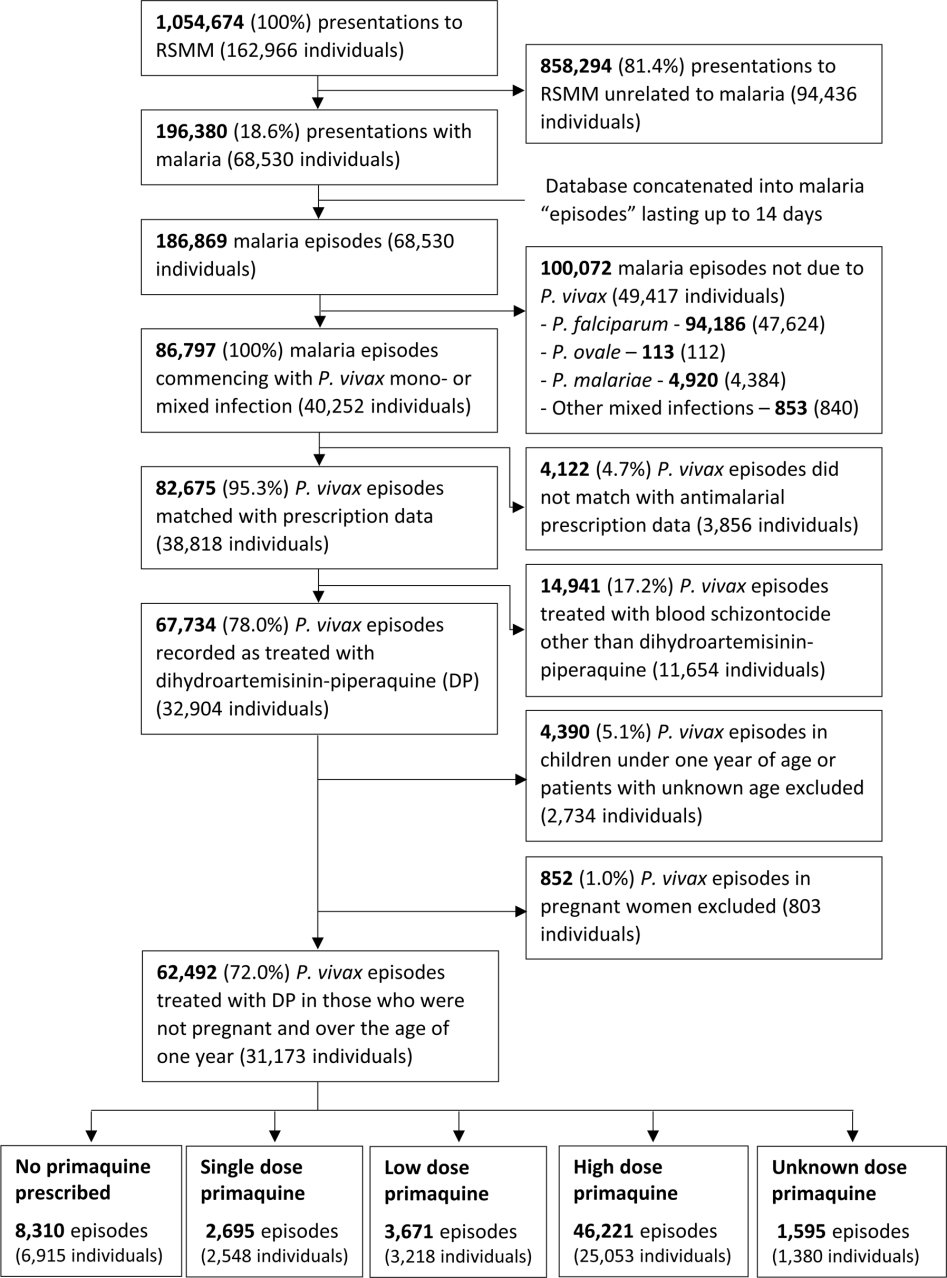
Study profile.

Between April 2004 and December 2013, there were 1,054,674 presentations to Mitra Masyarakat Hospital made by 162,966 individuals. The exclusion of presentations without malaria and concatenation of the dataset left 186,869 malaria episodes, of which 86,797 (46.4%) were due to *P*. *vivax* infection, and 82,675 (95.3%) could be matched with pharmacy records (Fig 2).

**Fig 3:**
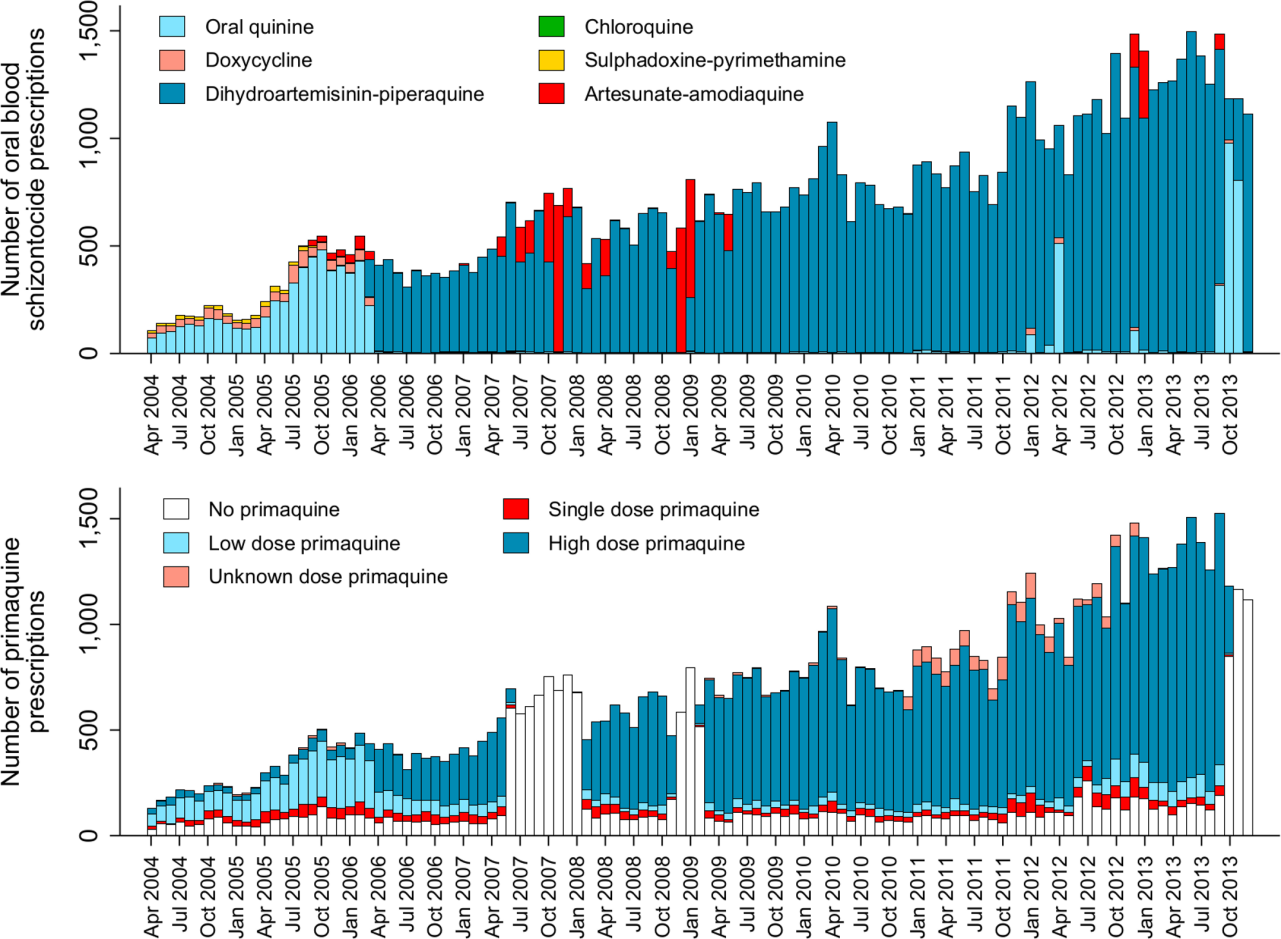
The absolute number of blood schizontocide and primaquine prescriptions at Mitra Masyarakat Hospital by year.

The total monthly prescriptions of oral antimalarials for patients with *P*. *vivax* infection are shown in Fig 3. There were 3 complete interruptions in the supply of primaquine, the largest from July 2007 to February 2008. After excluding pregnant women, children younger than 1 year of age and patients treated with non-DHP treatments, 62,492 (72.0%) clinical cases in 31,173 individuals, contributed to the primaquine effectiveness analysis (see Fig 2 and Table 1). Overall, 59.9%, 18.5%, and 21.7% of patients had 1, 2, or ≥3 (up to 24) episodes of vivax malaria during the study period, respectively.

**Table 1:** Distribution of demographic and clinical characteristics for patients included and excluded in the analysis.

	Included in the analysis	Excluded from the analysis
	N (%)	N (%)
**Initial species**		
Pure *P*. *vivax*	43,480 (69.6)	19,345 (79.6)
Mixed *P*. *vivax*	19,012 (30.4)	4,960 (20.4)
**Sex**		
Male	32,089 (51.3)	11,822 (48.6)
Nonpregnant females	30,403 (48.7)	11,069 (45.5)
Pregnant females	0 (0)	1,414 (5.8)
**Ethnicity**		
Non-Papuan	6,146 (9.8)	2,289 (9.4)
Highland Papuan	51,994 (83.2)	19,763 (81.3)
Lowland Papuan	4,318 (6.9)	2,239 (9.2)
**Age**		
Under 1 yr	-	5,823 (24.0)
1 to <5 yr	19,727 (31.6)	5,016 (20.6)
5 to <15 yr	13,155 (21.1)	3,130 (12.9)
≥15 yr	29,610 (47.4)	10,334 (42.5)
**Year**		
2004	0 (0)	2,272 (9.3)
2005	0 (0)	4,669 (19.2)
2006	3,147 (5.0)	1,894 (7.8)
2007	4,881 (7.8)	2,715 (11.2)
2008	5,396 (8.6)	1,872 (7.7)
2009	7,061 (11.3)	1,836 (7.6)
2010	8,591 (13.7)	1,033 (4.2)
2011	9,776 (15.6)	1,239 (5.1)
2012	11,488 (18.4)	2,571 (10.6)
2013	12,152 (19.4)	4,204 (17.3)
**Initial admission status**		
Outpatient	58,129 (93.0)	20,189 (83.1)
Inpatient	4,363 (7.0)	4,116 (16.9)
**Total**	62,492 (100)	24,305 (100)

Age data were missing for 2 patients. Ethnicity data were missing for 35 patients.

**Fig 4:**
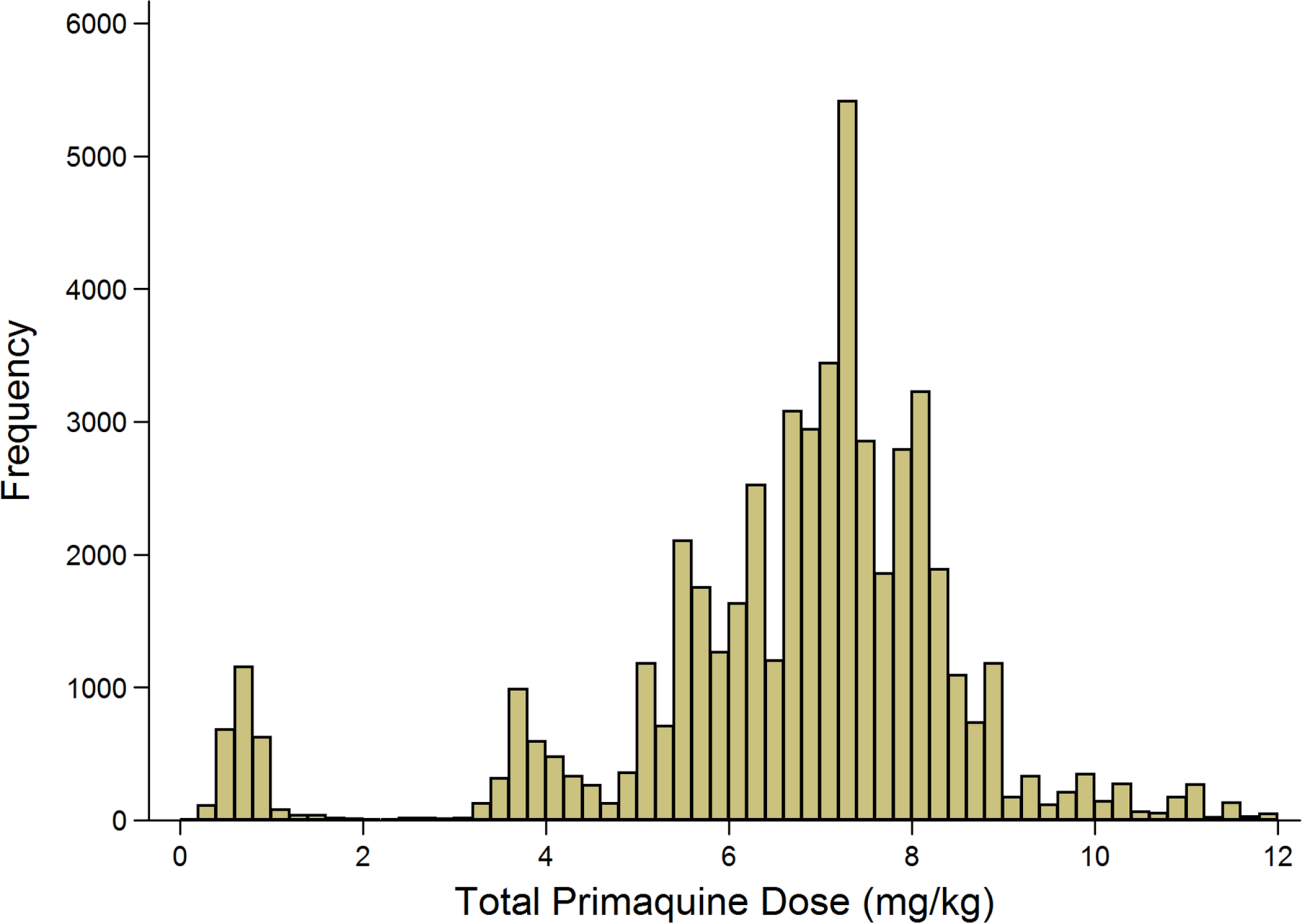
Histogram of the derived mg/kg total dose of primaquine administered.

A total of 86.7% (54,182/62,492) of *P*. *vivax* episodes were treated with primaquine, and the dose prescribed could be determined in 52,587 (97.1%) of these cases (Fig 4). Single-dose primaquine was prescribed in 2,695 (5.1%) of these vivax malaria episodes, a 14-day low-dose primaquine regimen in 3,672 (7.0%) episodes, and a 14-day high-dose primaquine regimen in 46,221 (87.9%) episodes. In total, 9.1% (335/3,672) of patients who were prescribed low-dose primaquine were predicted to have received less than 3.5 mg/kg of primaquine, and 39.8% (18,373/46,221) of patients prescribed a high dose regimen were administered a total dose less than 7 mg/kg. Patients receiving an unknown dose of primaquine were more likely to be less than 5 years old (72.4%, 1,154/1,595) compared to those with a known primaquine dose (30.5%, 18,573/60,897); Table 2. Patients receiving doses in the single-dose category were more likely to be adults than those receiving higher doses of primaquine (60.2% [1,622/2,695] versus 47.3% [21,883/46,221]). Other baseline characteristics are presented in Table 2.

**Table 2:** The distribution of risk factors for re-presentation with *P*. *vivax* malaria by primaquine dose category.

	No primaquine	Low-dose primaquine	High-dose primaquine	Single-dose primaquine	Unknown dose primaquine	Total
	N (%)	N (%)	N (%)	N (%)	N (%)	N (%)
**Initial species**						
Pure *P*. *vivax*	6,213 (74.8)	2,573 (70.1)	31,422 (68.0)	2,130 (79.0)	1,142 (71.6)	43,480 (69.6)
Mixed *P*. *vivax*	2,097 (25.2)	1,098 (29.9)	14,799 (32.0)	565 (21.0)	453 (28.4)	19,012 (30.4)
**Sex**						
Male	3,953 (47.6)	1,599 (43.6)	24,368 (52.7)	1,330 (49.4)	839 (52.6)	32,089 (51.3)
Female	4,357 (52.4)	2,072 (56.4)	21,853 (47.3)	1,365 (50.6)	756 (47.4)	30,403 (48.7)
**Ethnicity**						
Non-Papuan	933 (11.2)	481 (13.1)	4,478 (9.7)	218 (8.1)	36 (2.3)	6,146 (9.8)
Highland Papuan	6,809 (82.0)	2,955 (80.6)	38,579 (83.5)	2,255 (83.7)	1,396 (87.5)	51,994 (83.2)
Lowland Papuan	563 (6.8)	232 (6.3)	3,138 (6.8)	222 (8.2)	163 (10.2)	4,318 (6.9)
**Age**						
1 to <5 yr	3,274 (39.4)	355 (9.7)	14,414 (31.2)	530 (19.7)	1,154 (72.4)	19,727 (31.6)
5 to <15 yr	1,454 (17.5)	795 (21.7)	9,924 (21.5)	543 (20.1)	439 (27.5)	13,155 (21.1)
≥15 yr	3,582 (43.1)	2,521 (68.7)	21,883 (47.3)	1,622 (60.2)	2 (0.1)	29,610 (47.4)
**Year**						
2006	264 (3.2)	748 (20.4)	1,806 (3.9)	325 (12.1)	4 (0.3)	3,147 (5.0)
2007	2,977 (35.8)	265 (7.2)	1,445 (3.1)	192 (7.1)	2 (0.1)	4,881 (7.8)
2008	972 (11.7)	284 (7.7)	3,818 (8.3)	318 (11.8)	4 (0.3)	5,396 (8.6)
2009	960 (11.6)	314 (8.6)	5,476 (11.8)	256 (9.5)	55 (3.4)	7,061 (11.3)
2010	355 (4.3)	332 (9.0)	7,478 (16.2)	320 (11.9)	106 (6.6)	8,591 (13.7)
2011	318 (3.8)	405 (11.0)	7,831 (16.9)	370 (13.7)	852 (53.4)	9,776 (15.6)
2012	563 (6.8)	599 (16.3)	9,137 (19.8)	617 (22.9)	572 (35.9)	11,488 (18.4)
2013	1,901 (22.9)	724 (19.7)	9,230 (20.0)	297 (11.0)	0 (0.0)	12,152 (19.4)
**Initial admission status**						
Outpatient	7,528 (90.6)	3,292 (89.7)	43,431 (94.0)	2,457 (91.2)	1,421 (89.1)	58,129 (93.0)
Inpatient	782 (9.4)	379 (10.3)	2,790 (6.0)	238 (8.8)	174 (10.9)	4,363 (7.0)
**Total**	8,310 (100)	3,671 (100)	46,221 (100)	2,695 (100)	1,595 (100)	62,492 (100)

Ethnicity data were missing for 34 patients.

### Baseline risk factors for re-presentation with *P*. *vivax* infection

**Fig 5:**
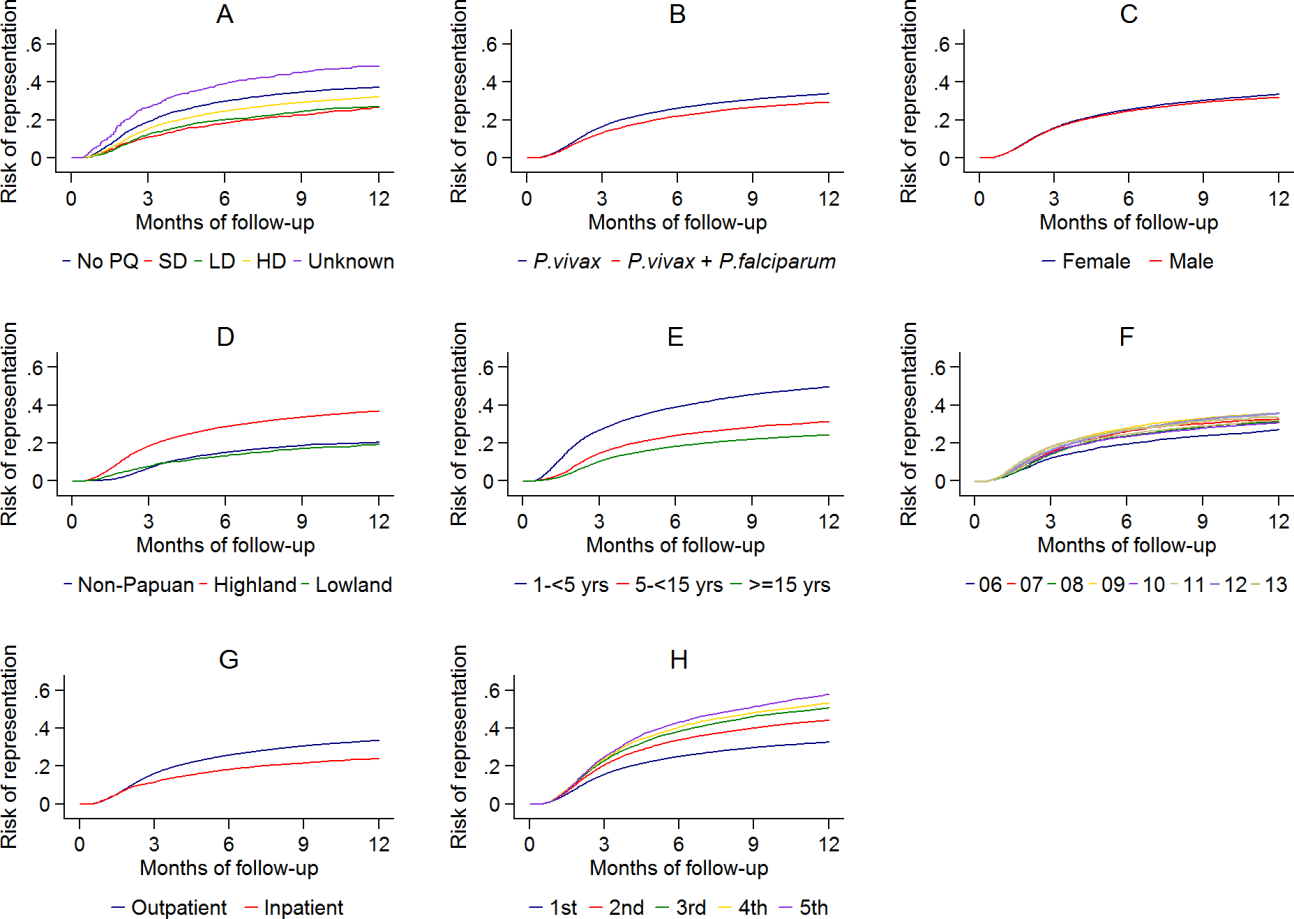
The cumulative risk of re-presentation with *P*. *vivax* malaria by baseline risk factor. A: primaquine dose category; B: *P*. *vivax* mono- or mixed infection; C: gender; D: ethnicity; E: age group; F: year; G: initial treatment as an outpatient or inpatient; H: vivax malaria episode number. (PQ, Primaquine; SD, Single Dose; LD, Low Dose; HD, High Dose).

The cumulative risk of re-presentation with clinical *P*. *vivax* infection within 1 year was 33.8% (95% Confidence Interval [95% CI] 33.1%–34.5%) after initial *P*. *vivax* monoinfection, and 29.2% (95% CI 28.1%–30.4%) after mixed-species infection (Fig 5 panel A). The rate of re-presentation reduced markedly with advancing age with children 1 to <5 years of age having an Adjusted Hazard Ratio (AHR) of 2.23 (95% CI 2.15–2.31) compared to patients 15 years of age or older (Table 3). The risk of re-presentation with vivax malaria was also lower in patients requiring admission to a hospital for treatment (23.9%, 95% CI 22.2%–25.8%) compared to those treated as outpatients (33.5% [95% CI 32.9%–34.2%]); AHR = 0.81 (95% CI: 0.76–0.86), *p* = <0.001. The greater the number of previous *P*. *vivax* infections an individual had had, the more likely they were to re-present with another *P*. *vivax* recurrence (Table 3; Fig 5 panel H).

**Table 3:** Baseline risk factors for re-presenting with *P*. *vivax* malaria after initial *P*. *vivax* malaria.

	Cumulative risk of re-presentation with vivax malaria	Univariable analysis		Multivariable analysis^b^	
		Crude HR [95% CI]	*p*	AHR [95% CI]	*p*
**Initial Species**^a^					
Pure *P*. *vivax*	33.8% [95%CI 33.1%–34.5%]	Reference		Reference	
Mixed *P*. *vivax/P*. *falciparum*	29.2% [95%CI 28.1%–30.4%]	0.83 [0.79–0.88]	<0.001	0.86 [0.83–0.88]	<0.001
**Gender**^a^					
Female	33.4% [95%CI 32.5%–34.3%]	Reference		Reference	
Male	31.8% [95%CI 31.0%–32.7%]	0.95 [0.90–0.99]	0.019	1·00 [0.97–1.03]	0.932
**Age**^a^^,^^c^					
1 to <5 yr	49.6% [95%CI 48.4%–50.9%]	2.54 [2.41–2.67]	<0.001	2.23 [2.15–2.31]	<0.001
5 to <15 yr	31.2% [95%CI 29.7%–32.7%]	1.35 [1.27–1.45]	<0.001	1.20 [1.15–1.26]	<0.001
≥15 yr	24.2% [95%CI 23.4%–24.9%]	Reference		Reference	
**Year**^a^					
2006	27.0% [95%CI 25.2%–28.8%]	0.85 [0.77–0.94]	0.002	0.85 [0.79–0.92]	<0.001
2007	32.4% [95%CI 30.6%–34.3%]	1.07 [0·98–1.18]	0.140	1.00 [0.93–1.07]	0.924
2008	31.2% [95%CI 29.3%–33.1%]	1.02 [0·92–1.12]	0.757	1.00 [0.94–1.06]	0.911
2009	35.8% [95%CI 34.0%–37.6%]	1.21 [1.11–1.33]	<0.001	1.13 [1.07–1.20]	<0.001
2010	30.6% [95%CI 29.0%–32.3%]	Reference		Reference	
2011	31.9% [95%CI 30.3%–33.5%]	1.05 [0.96–1.15]	0.248	1.04 [0.99–1.10]	0.143
2012	35.7% [95%CI 34.2%–37.3%]	1.20 [1.10–1.31]	<0.001	1.19 [1.13–1.26]	<0.001
2013	33.5% [95%CI 31.4%–35.7%]	1.18 [1.07–1.30]	0.001	1.21 [1.14–1.28]	<0.001
**Admission Status**^a^					
Outpatient	33.5% [95%CI 32.9%–34.2%]	Reference		Reference	
Inpatient	23.9% [95%CI 22.2%–25.8%]	0.68 [0.62–0.74]	<0.001	0.81 [0.76–0.86]	<0.001
**Presentation Number**					
1	32.6% [95%CI 32.0%–33.2%]	Reference		Reference	
2	44.1% [95%CI 43.2%–45.0%]	1.46 [1.41–1.51]	<0.001	1.35 [1.30–1.40]	<0.001
3	50.7% [95%CI 49.5%–51.8%]	1.74 [1.68–1.81]	<0.001	1.54 [1.48–1.61]	<0.001
4	53.3% [95%CI 51.9%–54.7%]	1.87 [1.79–1.95]	<0.001	1.63 [1.56–1.71]	<0.001
5	57.8% [95%CI 56.1%–59.5%]	2.06 [1.96–2.16]	<0.001	1.79 [1.71–1.89]	<0.001

Note: Patients treated with blood schizontocides other than dihydroartemisinin-piperaquine, pregnant women, and infants less than 1 year of age were excluded from all the models. Abbreviations: AHR, Adjusted Hazard Ratio; CI, Confidence Interval; HR, Hazard ratio

^a^ Univariable risks were derived from the first presentation with vivax malaria since subsequent presentations were not independent.

^b^ Cox model stratified by ethnicity (non-Papuan, Highland Papuan, and Lowland Papuan) and primaquine treatment regimen. Other co-variables: admission status (outpatient or inpatient), age group, gender, and presentation number. The variance–covariance matrices were corrected for intraindividual correlation using robust standard errors.

^c^ The median time to re-presenting with a vivax infection was 2.8 months for children less than 5 years of age compared with 3.4 months for 5 to <15 and 15 plus years.

### Primaquine effectiveness

The unadjusted cumulative risk of re-presentation with *P*. *vivax* malaria was 37.2% (95% CI 35.6%–38.8%) after no primaquine, 26.7% (24.1%–29.5%) after single-dose primaquine, 26.9% (24.8%–29.0%) after low-dose primaquine, 32.1% (31.4%–32.9%) after high-dose primaquine, and 48.3% (44.0%–52.9%) after unknown dose primaquine. Overall patients treated with any radically curative primaquine regimen (low dose or high dose) had a risk of re-presentation of 31.6% (95% CI 30.9%–32.3%) with an AHR for re-presentation with vivax malaria of 0.90 (95% CI 0.86%–0.95, *p* < 0.001) compared to patients who did not receive any primaquine (Table 4). Restricting the model to patients receiving either high-dose primaquine or no primaquine in the period during or 12 months before and after the largest primaquine stock outage did not alter the estimated protective effect of primaquine (AHR 0.91, 95% CI 0.85–0.97, *p* = 0.003). High-dose primaquine was associated with a 15% reduction in the rate of re-presentation compared with no primaquine in children less than 5 years of age (AHR 0.85, 95% CI 0.79–0.90, *p* < 0.001), whereas no significant effect was seen in 5 to <15 year olds (AHR 0.98, 95% CI 0.86–1.12, *p* = 0.76) and those ≥15 years of age (AHR 0.99, 95% CI 0.91–1.08, *p* = 0.88). To reduce potential confounding due to reinfection, the overall effectiveness and subgroup analyses were restricted to 3 months of follow-up; the results were similar (Table 5).

**Table 4:** Results of Cox proportional hazards models for the effect of primaquine on the risk of re-presentation to hospital with *P*. *vivax* malaria within 12 months of being treated for *P*. *vivax* malaria.

	Primaquine dose	AHR	95% Confidence interval	*p*
**Overall model**				
All^a^	None	1.00*	–	–
	Low- or high-dose Primaquine	0.90	0.86–0.95	<0.001
**Subgroup analyses**				
1 to <5 yr^b^	None	1.00*	–	–
	≥5 mg/kg (high dose)	0.85	0.79–0.90	<0.001
5 to <15 yr^b^	None	1.00*	–	–
	≥5 mg/kg (high dose)	0.98	0.86–1.12	0.764
≥15 yr^b^	None	1.00*	–	–
	≥5 mg/kg (high dose)	0.99	0.91–1.08	0.876
2007^c^	None	1.00*	–	–
	≥5 mg/kg (high dose)	0.91	0.85–0.97	0.003
Primaquine Dose ≥7 mg/kg^a^	None	1.00*		
	≥7 mg/kg	0.91	0.86–0.96	<0.001
Outpatients^d^	None	1.00*	–	–
	≥5 mg/kg (high dose)	0.89	0.84–0.93	<0.001

Note: Patients treated with blood schizontocides other than dihydroartemisinin-piperaquine, pregnant women, and infants less than 1 year of age were excluded from all the models. Abbreviations: AHR, Adjusted Hazard Ratio.

* Reference category.

^a^ Cox model stratified by ethnicity (non-Papuan, Highland Papuan, and Lowland Papuan) and age group (1 to <5 yr, 5 to <15 yr, and ≥15 yr). Other co-variables include gender, year, admission status (outpatient or inpatient), and presentation number.

^b^ Cox models stratified by ethnicity. Other co-variables include gender, year, admission status, and presentation number. Age group was found to be an effect modifier of the association between primaquine dose and vivax recurrence; *p* < 0.001 from likelihood ratio test comparing models with and without an interaction term between age group and primaquine dosing.

^c^ Model limited to period during and 12 months before and after the largest primaquine stock outage in 2007. Cox model stratified by ethnicity and age group. Other co-variables include gender, admission status, and presentation number.

^d^ Cox model stratified by ethnicity and age group. Other co-variables include gender, year, and presentation number.

**Table 5:** Results of Cox proportional hazards models for the effect of primaquine on the risk of re-presentation to hospital with *P*. *vivax* malaria within 3 months of being treated for *P*. *vivax* malaria.

	Primaquine dose	AHR	95% Confidence interval	*p*
**Overall model**				
All^a^	None	1.00*	–	–
	Low- or high-dose primaquine	0.88	0.82–0.94	<0.001
**Subgroup analyses**				
1 to <5 yr^b^	None	1.00*	–	–
	≥5 mg/kg (high dose)	0.82	0.75–0.89	<0.001
5 to <15 yr^b^	None	1.00*	–	–
	≥5 mg/kg (high dose)	0.94	0.77–1.16	0.579
≥15 yr^b^	None	1.00*	–	–
	≥5 mg/kg (high dose)	1.03	0.90–1.17	0.672
2007^c^	None	1.00*	–	–
	≥5 mg/kg (high dose)	0.82	0.75–0.91	<0.001
Primaquine dose ≥7 mg/kg^a^	None	1.00*		
	≥ 7 mg/kg	0.88	0.82–0.95	0.001
Outpatients^d^	None	1.00*	–	–
	≥5 mg/kg (high dose)	0.86	0.81–0.93	<0.001

Note: Patients treated with blood schizontocides other than dihydroartemisinin-piperaquine, pregnant women, and infants less than 1 year of age were excluded from all the models. Abbreviations: AHR, Adjusted Hazard Ratio

* Reference category.

^a^ Cox model stratified by ethnicity (non-Papuan, Highland Papuan, and Lowland Papuan) and age group (1 to <5 yr, 5 to <15 yr and ≥15 yr). Other co-variables include gender, year, admission status (outpatient or inpatient), and presentation number.

^b^ Cox models stratified by ethnicity. Other co-variables include gender, year, admission status, and presentation number. Age group was found to be an effect modifier of the association between primaquine dose and vivax recurrence; *p* < 0.001 from likelihood ratio test comparing models with and without an interaction term between age group and primaquine dosing.

^c^ Model limited to the period during and 12 months before and after the largest primaquine stock outage in 2007. Cox model stratified by ethnicity and age group. Other co-variables include gender, admission status, and presentation number.

^d^ Cox model stratified by ethnicity and age group. Other co-variables include gender, year, and presentation number.

## Discussion

We present a large-scale comparative assessment of the effectiveness of unsupervised primaquine for preventing recurrent *P*. *vivax* malaria in a nontrial setting. Despite good staff adherence to the WHO-endorsed dosing regimen, prescription of primaquine at RSMM was associated with, at best, a modest reduction of approximately 10% in the rate of re-presentation to hospital with vivax malaria within 1 year.

Recurrent *P*. *vivax* malaria has important implications both for individuals and populations in endemic regions [22], resulting in cumulative anemia due to repeated episodes of hemolysis, impaired nutrition, poor cognitive performance, and school absenteeism [12, 23]. Furthermore, at a community level, recurrent infections are an important source of transmission and the main barrier to elimination of this parasite [24–26]. Safe and effective radical cure of *P*. *vivax* is therefore an essential component of optimal treatment guidelines. Our analysis suggests that the strategy of long, unsupervised treatment courses of primaquine is compromised significantly in this resource-poor setting.

Our pragmatic study has a number of important strengths. Due to very large numbers, our estimates of the risk of re-presentation are unlikely to be attributable to chance. Hospital policy dictates that all patients presenting with fever are offered laboratory tests for the diagnosis of malaria. Microscopy services at RSMM have performed well in quality assurance procedures [15]; thus, parasitological diagnoses are comparatively accurate when considered in the context of field laboratory services. Crucially, patients included in our analyses received their medications according to normal hospital procedures and were free from the powerful biases and clinical influences associated with many study designs [9]. Although this is an important strength, it is also a notable limitation. In the absence of randomized and contemporaneous allocation, our data are susceptible to the effects of residual bias due to unknown confounders. Restriction of the effectiveness model to the period during and 12 before and after the largest primaquine stock outage was intended to reduce confounders associated with the clinical decision to give or withhold primaquine. Importantly, this model did not substantially change the estimate of the effectiveness of primaquine, suggesting minimal residual confounding in the overall model.

The potential explanations for the modest observed effectiveness of primaquine can be divided broadly into those pertaining to the host, the parasite, the drug, or the study design. Poor adherence to an unsupervised 14-day treatment regimen is likely to be a significant host factor. It is well recognized that the duration of antimalarial treatment is a critical determinant of adherence [9]. The 14-day primaquine regimen currently recommended in the WHO antimalarial treatment guidelines is more than 4 times longer than most schizontocidal regimens. Since the symptoms of malaria usually subside within 2 or 3 days of initiation of treatment, the incentive to take all doses of primaquine is low. Recent randomized controlled trials conducted in Thailand and Ethiopia demonstrated that directly observed primaquine therapy was associated with a 3-to-4-fold lower rate of vivax recurrence when compared to unsupervised therapy [7, 27]. In our study, primaquine effectiveness was highest in young children, and this may reflect parental encouragement and supervision of tablet administration. Another host factor could be a reduced ability to metabolise primaquine to its active metabolite. Recent evidence has implicated polymorphisms in the cytochrome (CYP)450 2D6 gene in the treatment failure of patients following primaquine therapy [28]. However, whilst these genetic variants have been documented across most of the globe, they usually occur in about 10% of the population [29], and thus would be unlikely alone to account for high rates of treatment failure in our analysis.

Tolerance or resistance of the parasite to primaquine could have played a role. Although strains of *P*. *vivax* from Southeast Asia and the Pacific, and, in particular, the Chesson strain of the Island of New Guinea, are thought to be relatively unresponsive to primaquine and other 8-aminoquinoline compounds [30, 31], a recent study of Indonesian soldiers with *P*. *vivax* acquired in Papua Province demonstrated that a supervised regimen of DHP plus a high-dose primaquine regimen had excellent efficacy [32]. Significant primaquine resistance or tolerance in this region therefore seems unlikely.

Poor quality and/or counterfeit antimalarials have been recognized as a substantial threat to malaria-control programs, generally, and a catalyst for the propagation of multidrug-resistant parasites [33–35]. Although poor-quality primaquine could have contributed to the poor effectiveness observed in this study, previous studies in Papua of supervised primaquine regimens, with a drug from the same manufacturer showed excellent prophylactic efficacy, so this is unlikely to have been a major contributor to our findings [32, 36, 37]. Further studies are underway to confirm the quality of the primaquine tablets currently available in Timika; however; the possibility of significant batch to batch variation over the 8 years of the study cannot be ruled out. Alternatively, the observed poor effectiveness could have arisen from low-primaquine concentrations due to patients being underdosed. There was a wide range in the total mg/kg primaquine dose prescribed, with almost 40% of those prescribed the high-dose regimen receiving a dose below the 7 mg/kg target dose. However, in a subgroup analysis in which patients receiving a total dose of primaquine greater than 7 mg/kg were compared with those prescribed no primaquine, the adjusted hazards ratio was unchanged (Table 4), suggesting that underdosing alone did not account for primaquine’s poor effectiveness (Table 4).

Our observational study design and passive follow-up will have resulted in attrition bias. Previous clinical trials in this location have shown that recurrent *P*. *vivax* parasitemia can be detected in up to 40% of patients by day 42 after schizontocidal treatment alone, with a high proportion of these recurrences being asymptomatic [17, 38]. In our hospital-based surveillance, only a third of patients re-presented to hospital with *P*. *vivax* malaria within a year, potentially reflecting less severe or asymptomatic recurrent infections, seeking treatment at alternative facilities, or migration out of the area. Since our study was not designed to identify asymptomatic relapses, which are a major source of transmission potential [26], we cannot rule out the possibility that unsupervised primaquine may have had a significant public health benefit through the reduction of the asymptomatic parasite reservoir [39].

In summary, our study highlights, at best, a modest benefit of unsupervised high-dose primaquine for preventing clinical relapses of vivax malaria in southern Papua. The elimination of *P*. *vivax* will require practicable, safe, and effective radical cure of both blood and liver stage infections. Building stronger health systems, educating patients on the importance of treatment adherence, and supervision of therapy will be critical for improving the outcomes of primaquine radical cure for *P. vivax* [1, 40]. However, ultimately, a hypnozoitocidal treatment regimen is needed, which is more amenable to high prescriber and patient adherence than the current prolonged, and potentially toxic, course of primaquine. Radical cure has been achieved with a single dose of tafenoquine, and this drug is now in phase III clinical trials [41]. Until tafenoquine or an alternative agent is licensed and widely available, public health measures aimed at increasing the adherence to primaquine regimens, either through supervision or more practical dosing regimens, need to be implemented as a matter of urgency.

## Supporting information

S1 Text Statistical plan. (DOCX)

S2 Text RECORD checklist. (DOCX)

## Abbreviations

AHR: adjusted hazard ratio
DHP: Dihydroartemisinin-piperaquine
G6PD: glucose-6-phosphate dehydrogenase
HRP2: Histidine Rich Protein-2
RSMM: Rumah Sakit Mitra Masyarakat
WHO: World Health Organization
