# Supplementary material for: Unsupervised primaquine for the treatment of Plasmodium vivax malaria relapses in southern Papua: A hospital-based cohort study
Source: PLoS Med. 2017 Aug 29;14(8):e1002379. doi: 10.1371/journal.pmed.1002379 (PMC5574534; doi:10.1371/journal.pmed.1002379)
Supplement: S1 Text — (DOCX) [file pmed.1002379.s001.docx]

**Supplementary File 1**

**Effectiveness of unsupervised primaquine for the treatment of *Plasmodium vivax* relapses in southern Papua: a hospital-based cohort study**

**Analytical Plan**

**Research Question**

Does unsupervised primaquine, prescribed in combination with dihydroartemisinin-piperaquine, for *Plasmodium vivax* infection reduce the risk of a further presentation to hospital with *P. vivax* infection within one year in southern Papua.

**Outcome of Interest**

Risk of representation to hospital with *P. vivax* infection within one year of initial presentation to hospital with *P. vivax* infection.

**Study Population**

Patients presenting to any department at Rumah Sakit Mitra Masyarakat with microscopically confirmed *P. vivax* parasitaemia (either as a monoinfection or part of a mixed infection) regardless of presence or absence of symptoms.

**Exclusions**

- Clinical episodes not associated with parasitaemia or associated with infection by *Plasmodium* species other than *P. vivax.*
- Clinical episodes not matched with antimalarial prescription records.
- Clinical episodes of *P. vivax* infection treated with blood schizontocidal drugs other than dihydroartemisinin-piperaquine.
- Clinical episodes in infants under the age of one year.
- Clinical episodes in patients without age data.
- Clinical episodes in pregnant women in any trimester.

**Primaquine Dose Categories**

1. No primaquine (matching antimalarial prescription data but no primaquine prescribed)
2. Single dose primaquine (>0mg/kg and <1.5mg/kg total dose)
3. Low dose primaquine (≥1.5mg/kg and <5mg/kg total dose)
4. High dose primaquine (≥5mg/kg total dose)
5. Unknown dose primaquine (matched primaquine prescription record but unable to determine dose in mg per kg)

**Follow-up**

- Multiple presentations with *Plasmodium* parasitaemia within 14 days concatenated into a single clinical event.
- Follow-up to be truncated at the time of death or the 31^st^ December 2013.

**Independent Factors**

- Age (categorised as 1 to <5 years, 5 to <15 years, ≥15 years)
- Gender
- Ethnicity: Non-Papuan, Highland Papuan, Lowland Papuan.
- Presence or absence of a second or third *Plasmodium* species (ie *P. vivax* monoinfection or mixed infection).
- Year of presentation (2006 through to 2013).
- Inpatient or outpatient status (on the day of initial presentation).
- Number of previous presentations with *P. vivax* infection (up to and including five).

**Statistical Analysis Software**

STATA version 12.1

**Statistical Analysis Methods**

- Primary comparison to be between patients receiving low dose or high dose primaquine versus those receiving no primaquine.
- Prespecified subgroup comparisons (all limited to high dose primaquine versus no primaquine):
  - Each age category (1 to <5 years, 5 to <15 years, ≥15 years).
  - During and 6 months either side of largest primaquine stock outage in 2007.
  - Patients initially treated as outpatients.
  - Patients receiving therapeutic high dose primaquine (total dose >7mg/kg) – ***(added in response to reviewers comments)***.
- Kaplan-Meier analysis for risk of representation with *P. vivax* infection. Includes time from first to second presentation only. The duration of the survival analysis to be determined by the time at which the background risk of recurrence reaches steady state.
  - Prespecified sensitivity analysis: Comparison between patients receiving low dose or high dose primaquine versus those receiving no primaquine with follow-up truncated at 3 months instead of one year – to reduce the confounding effect of new infections compared to relapsing infections.
- Univariable followed by multivariable Cox regression analysis for risk of representation with *P. vivax* infection within one year. Includes up to 5 presentations per individual.
- Proportional hazards assumption to be tested using log(cumulative hazard) by time of follow-up curves and by fitting and comparing models with and without time interaction terms by means of the likelihood ratio test.
- Multivariable models to be stratified by any covariables found to violate proportional hazards assumption.
- Intra-patient correlation to be allowed for in both univariable and multivariable Cox regression models by calculating robust standard errors (Huber-White sandwich estimator).
